# Repurposing the FDA-Approved Pinworm Drug Pyrvinium as a Novel Chemotherapeutic Agent for Intestinal Polyposis

**DOI:** 10.1371/journal.pone.0101969

**Published:** 2014-07-08

**Authors:** Bin Li, Colin A. Flaveny, Camilla Giambelli, Dennis Liang Fei, Lu Han, Brian I. Hang, Feng Bai, Xin-Hai Pei, Vania Nose, Oname Burlingame, Anthony J. Capobianco, Darren Orton, Ethan Lee, David J. Robbins

**Affiliations:** 1 Molecular Oncology Program, Department of Surgery, University of Miami, Miami, Florida, United States of America; 2 Sylvester Comprehensive Cancer Center, University of Miami, Miami, Florida, United States of America; 3 Department of Biochemistry and Molecular Biology, University of Miami, Miami, Florida, United States of America; 4 Department of Pathology, Miller School of Medicine, University of Miami, Miami, Florida, United States of America; 5 Department of Pathology, Jackson Health System, University of Miami, Miami, Florida, United States of America; 6 Department of Cell and Developmental Biology, Vanderbilt University School of Medicine, Nashville, Tennessee, United States of America; 7 Stemsynergy Therapeutics Inc., Miami, Florida, United States of America; Baylor college of Medicine, United States of America

## Abstract

Mutations in the WNT-pathway regulator ADENOMATOUS POLYPOSIS COLI (APC) promote aberrant activation of the WNT pathway that is responsible for APC-associated diseases such as Familial Adenomatous Polyposis (FAP) and 85% of spontaneous colorectal cancers (CRC). FAP is characterized by multiple intestinal adenomas, which inexorably result in CRC. Surprisingly, given their common occurrence, there are few effective chemotherapeutic drugs for FAP. Here we show that the FDA-approved, anti-helminthic drug Pyrvinium attenuates the growth of WNT-dependent CRC cells and does so via activation of CK1α. Furthermore, we show that Pyrvinium can function as an *in vivo* inhibitor of WNT-signaling and polyposis in a mouse model of FAP: *APC^min^* mice. Oral administration of Pyrvinium, a CK1α agonist, attenuated the levels of WNT-driven biomarkers and inhibited adenoma formation in *APC^min^* mice. Considering its well-documented safe use for treating enterobiasis in humans, our findings suggest that Pyrvinium could be repurposed for the clinical treatment of APC-associated polyposes.

## Introduction

The WNT-signaling pathway plays a pivotal role in embryonic development, stem cell biology, maintenance of the normal intestinal epithelium, and ultimately as a driver of carcinogenesis. In the absence of WNT activity, steady-state levels of the transcriptional activator β-CATENIN (CTNNB1) are reduced by a destruction complex consisting of ADENOMATOUS POLYPOSIS COLI (APC), GLYCOGEN SYNTHASE-KINASE 3β (GSK3β), CASEIN KINASE-1 α (CK1α) and AXIN [1]–[4]. GSK3β and CK1α phosphorylate CTNNB1 at specific serine and threonine residues leading to its recognition by the F-box protein β-TRCP and eventual proteasomal degradation [1]–[3], [5]–[7]. Upon WNT activation the destruction complex is disassembled, CTNNB1 is stabilized and accumulates in the nucleus where WNT-target gene expression is elevated leading to proliferation and growth [1]–[3], [5]. WNT-pathway activation is a key factor in the etiology and maintenance of colorectal cancer (CRC), with loss of function mutations in the tumor suppressor *APC* being the main cause [8]–[10]. Truncated *APC* mutants and degradation resistant *CTNNB1* point mutations are found in 80% and 10% of all spontaneous CRC cases respectively [10], [11]. In spontaneous CRC, alterations in *APC* mark the earliest event leading to carcinogenesis whereas mutations in other CRC associated oncogenes including *RAS* and the tumor suppressor *p53* are considered late events [11]. CRC cells are dependent on WNT signaling at the initiation stage of the disease and at later stages when WNT signaling is required to maintain a growth advantage, inhibiting differentiation and promoting stem cell expansion [12]–[16]. Therefore effective inhibition of activated WNT-signaling is a theoretically viable chemotherapeutic strategy for *APC*-associated polyposes and associated CRC.

*APC*-associated polyposes include Familial Adenomatous Polyposis (FAP), attenuated FAP and Turcot syndrome are all caused by germline loss of function mutations in the WNT-pathway repressor, *APC* [8], [9], [11], [17], [18]. These conditions are characterized by the formation of multiple abnormal tissue growths called adenomatous polyps along the inner intestinal walls. Typically FAP patients develop hundreds to thousands of precancerous adenomas at an early age, which if untreated will develop into CRC. As with most rare diseases, due to the small number of individuals affected, there is reduced incentive within the pharmaceutical industry to develop new drug treatments for *APC*-associated polyposis. Therefore for orphan diseases like FAP, effective drugs often have to be sourced from previously FDA-approved drugs. Current FAP treatment entails colectomy followed by regular screenings, however colectomy does not prevent extra-intestinal tumors, colon-stump tumors and other tumors of the intestines that can occur in FAP patients [19]–[25]. Few advances have been made in the treatment of pre-symptomatic and post-colectomy patients. Due to their ability to induce apoptosis in cultured CRC cells a number of non-steroidal anti-inflammatories (NSAIDs) have been tested as chemotherapeutic agents for CRC [26]–[29]. Sulindac and celecoxib are the only FDA approved NSAIDs for treating *APC*-associated polyposis, but these drugs have been used with limited success and prolonged use is accompanied by pernicious cardiovascular and gastrointestinal complications. Neither compound has been shown to reduce tumor size or clinically prevent tumor formation in FAP [26], [30], [31]. Furthermore, although a number of putative targets for NSAIDs in the intestinal epithelium have been proposed, including *CTNNB1*, *RAS, NFκB* and *PPARδ*, *in vivo* evidence for these targets have been lacking [27], [32], [33]. There is thus a critical need to identify chemotherapeutic agents for APC-associated polyposes that effectively block the pathology of these diseases *in vivo*.

In both *APC*-associated polyposis and CRC, WNT-activation promotes the transformation of normal colorectal mucosa to adenoma, then subsequent carcinoma following additional somatic mutations. As activated WNT signaling is also required for CRC viabiity, chemotherapeutic agents that target WNT signaling should have dual utility as inhibitors of polyposis and carcinogenesis in FAP. The FAP mouse model (*APC^min^* mice) has been used extensively to assess the efficacy of chemotherapeutic agents for the treatment of FAP and CRC [16], [30], [34], [35]. A number of genes that display elevated expression in *APC^min^* mice are also analogously upregulated in cultured CRC cells. Therefore *APC^min^* mice are a versatile model for studying the factors influencing the pathology of FAP, and so provide a vital mouse model for gauging the efficacy of novel chemotherapeutic agents for FAP.

We recently demonstrated that the FDA approved anti-helminthic drug Pyrvinium is able to attenuate WNT signaling [36], [37], through direct binding to and activation of CK1α [36]. Other studies have highlighted the emerging role of CK1α in regulating intestinal epithelial cell proliferation and inhibiting colorectal cancer progression [38], [39]. Further, it has been shown that expression of *CK1α* inhibits tumor invasion and metastasis [38]. In this study we evaluated the efficacy of Pyrvinium inhibiting WNT-signaling, via activating CK1α, in both CRC cells *in vitro* and in the intestinal epithelium of *APC^min^* mice. Pyrvinium treatment suppressed intestinal WNT activation and significantly reduced the numbers of intestinal polyps compared to vehicle treated mice. This study demonstrates the potential utility of CK1α activators as WNT-inhibitors in the treatment of WNT-driven diseases like *APC*-associated polyposis, and provides compelling pre-clinical data to justify repurposing Pyrvinium for FAP patients in future clinical studies.

## Materials and Methods

### Chemical compound information

**Figure 1:**
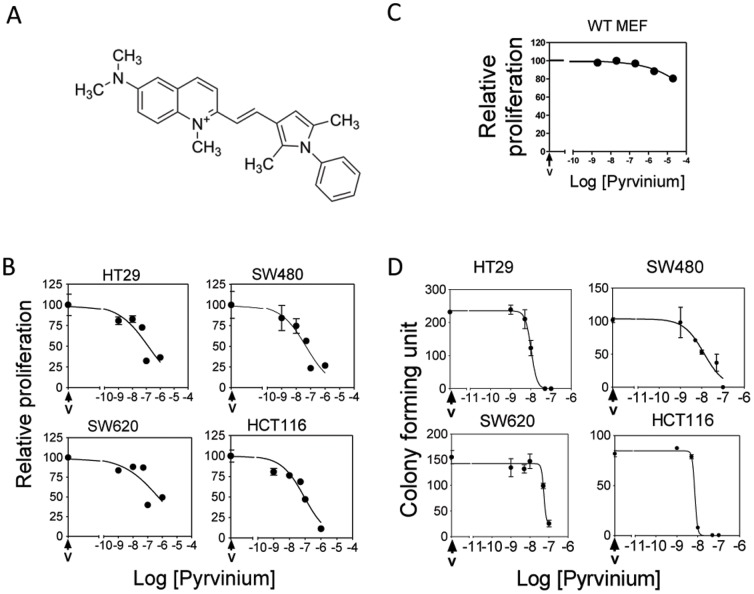
Pyrvinium reduces CRC cell viability. **A**. Structure of Pyrvinium. **B**. MTT assay of various CRC cells treated with increasing amounts of Pyrvinium or vehicle control (**V**). Treated plates were incubated with 10% MTT, lysed and MTT reduction assessed. **C**. Similar assay as B was performed using WT MEF cells. **D**. Clonogenic assay of Pyrvinium treated CRC cell lines. The CRC cell lines SW480, SW620, HT29 and HCT116 were treated with increasing concentrations of Pyrvinium or vehicle control for 2 weeks. Colonies were fixed with gluteraldehyde and stained with crystal violet. Mean number of colonies was quantitated relative to vehicle controls. Error bars indicate ± S.E.M of three experiments.

Pyrvinium pamoate (Pyrvinium) salt; 6-(Dimethylamino)-2-[2-(2,5-dimethyl-1-phenyl-1H-pyrrol-3-yl)ethenyl]-1-methyl-4,4′-methylenebis[3-hydroxy-2-naphthalenecarboxylate] (2∶1)-quinolinium. (see Fig 1A for structure) (Sigma-Aldrich). The cellular stress inducer Tunicamycin was purchased from Sigma-Aldrich. BKM120 was purchased from Selleck Chemicals.

### Cell-culture and cell based assays

All cells were obtained from ATCC except for HCT116 WTKO (in which the WT copy of *CTNNB1* has been deleted), which were a gift from Dr. Bert Vogelstein (Johns Hopkins University) [40]. Cells were cultured under standard conditions, 37°C at 5% CO_2_/95% air. HT29, SW620 and SW480 cells were grown in Dulbecco's-Minimum Essential Media (D-MEM) and HCT116 cells (which retains the WT copy of *CTNNB1*) were cultured in Roswell Park Memorial Institute Media (RPMI). All media was supplemented with 10% fetal bovine serum, and 100 IU/ml penicillin/100 µg/ml streptomycin. The clonogenic and MTT assays were performed as previously described [41], [42]. Luciferase activity was determined using Steady-Glo (Promega) according to manufacturer's guidance. Statistical significance was calculated using Student's t test. p<0.05 was considered statistically significant and marked with an asterisk.

### RT-PCR

For all qPCR experiments 2 µg of purified RNA was converted to cDNA using a cDNA synthesis kit (ABI). cDNA was then subjected to Taqman Real-Time qPCR using the cognate probes, qPCR master mix (Biorad) and PCR conditions as per the manufacturer's instructions (Invitrogen).

### XBP-1 splicing assay

Cells were treated with vehicle control, 100 nM Pyrvinium, 1 µg/ml Tunicamycin or both 100 nM Pyrvinium and 1 µg/ml Tunicamycin for 24 h. Total RNA was isolated and subjected to a semi-quantitative RT-PCR. The PCR product was then resolved using a 3% agarose gel. The size of the PCR product for a full length XBP-1 transcript was ∼380 bp and ∼350 bp for the active XBP-1S splice variants. The following primers were designed to distinguish between the full length and active short splice variant of XBP-1; XBP-1F: 5′-ccagagatcgaaagaaggctcgaat-‘3 and XBP-1R: 5′-gactgggtccaagttgtccagaa-3′.

### Immunoblotting

Total protein isolated from cells was heat denatured in Laemmli buffer, resolved by SDS-PAGE and transferred to PVDF membrane. These membranes were then probed for specific proteins using the cognate antibodies for CASEIN KINASE 1α (CK1α) (Santa Cruz Biotechnology), CYCLIN D1 (CCND1) (Cell Signaling), PYGOPUS2 (Abcam), CTNNB1 (Cell Signaling), S45 phospho-CTNNB1 (Cell Signaling), GAPDH (Abnova), β-ACTIN (Santa Cruz Biotechnology) and α-TUBULIN (Sigma). Nuclear extraction was performed using the NE-PER Nuclear and Cytoplasmic Extraction kit (Pierce).

### Animal model and ethics statement

This study was carried out in strict accordance with the recommendations in the Guide for the Care and Use of Laboratory Animals of the National Institutes of Health. The protocol was approved by Institutional Animal Care and Use Committee of the University of Miami (protocol# 10-237). All *APC^min^* mice (C57BL/6J-*Apc^Min^*/J) were obtained from Jackson Laboratories. These mice were housed in sterile ventilated cages, fed a standard diet, and provided water *ad libitum*. After two weeks acclimation mice were treated with Pyrvinium (25 mg/kg) or DMSO-vehicle control via oral gavage every 48 h or as otherwise specified. Mice were monitored daily for signs of illness, pain, or severe weight loss. All mice were euthanized using CO_2_ followed by cervical dislocation.

The entire intestinal tract was removed from euthanized mice flushed with PBS then fixed in 10% neutral buffered formalin. The isolated intestines from each mouse were assessed for gross polyp formation using a stereomicroscope. Fixed total intestinal tissue was embedded in paraffin cut into 4 µm thick sections and assessed for neoplasms. In situ TUNEL assays were performed using the DeadEnd colorimetric TUNEL assay as per manufacturer's instructions (Promega). Intestinal tissue sections were probed for protein expression as per manufacturer's instructions using anti-CTNNB1 antibody (Cell Signaling). Tissues were isolated from euthanized mice. Total mRNA was isolated from tissues homogenized in Trizol (Invitrogen) and purified using an RNA purification kit (Qiagen).

## Results

### Pyrvinium inhibits CRC cell viability

Pyrvinium (Fig 1A) significantly reduces CRC cell viability and growth as measured by both an MTT-reduction assay and a clonogenic growth assay, each of which quantifies cell viability (Fig 1 and Table 1). In MTT assays Pyrvinium treatment at nanomolar concentrations reduced proliferation of the CRC cell lines (Fig 1B and Table 1). Pyrvinium does not have a general toxicity on cell growth, as its ability to attenuate the proliferation of CRC cells lacking *APC* can be reduced by re-expressing *APC* [36]. In addition, Pyrvinium's ability to attenuate the proliferation of mouse embryonic fibroblasts (MEF) was significantly reduced (IC_50_∼2.5 mM) relative to its effects on CRC cells (Fig 1C). Pyrvinium also potently inhibited CRC cell colony formation (Fig 1D and Table 1; Fig S1). These results show that Pyrvinium can attenuate the growth of transformed intestinal cells harboring an aberrantly activated WNT-signaling pathway. Further, this result demonstrates Pyrvinium's growth inhibitory effect on CRC cells containing mutant *p53*, *RAS* and overexpressed *MYC* [43], all of which are present within the various CRC cell lines used (Table 1) and commonly found in FAP-associated CRC [44]–[47].

**Table 1:** Pyrvinium treated CRC cell lines: summary.

CRC Cells	MTT Assay IC_50_ (nM)	Clonogenic Assay IC_50_ (nM)	*P53* Mutations	Oncogenes Expressed
HT29	135	10	G>A (Arg->His) 273	*MYC, RAS, MYB, FOS*
SW480	47	13	G>A (Arg->His) 273/C>T (Pro->Ser) 309	*MYC, RAS, MYB, FOS*
SW620	513	54	G>A (Arg->His) 273	*MYC, RAS, MYB, FOS*
HCT116	87	8	WT p53, p21 deficient	*MYC, RAS, MYB*

### Pyrvinium attenuates WNT signaling in a CK1α dependent manner

**Figure 2:**
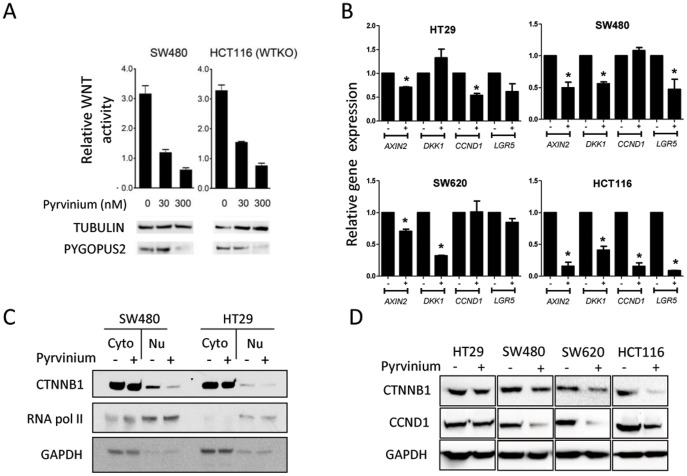
Pyrvinium inhibits WNT signaling in CRC cells. **A**. Pyrvinium inhibits WNT reporter gene activity in CRC cells. Cells were treated with Pyrvinium at the indicated concentrations and relative WNT reporter luciferase activity determined. Immunoblots show that Pyrvinium decreases PYGOPUS2 levels in both SW480 and HCT116 WTKO cells in a dose-dependent manner. Error bars indicate ± S.E.M of three experiments. **B**. Expression of WNT regulated genes *AXIN2*, *DKK1*, *CCND1* and *LGR5* in CRC cell lines treated with 100 nM Pyrvinium or vehicle control was determined using real-time RT-PCR. Error bars indicate ± S.E.M of three experiments. *p<0.05. **C**. A nuclear or cytosolic fraction was extracted from vehicle or 100 nM Pyrvinium treated SW480 and HT29 cells and CTNNB1 protein levels were determined by immunoblot. **D**. Immunoblot of the WNT associated biomarkers CTNNB1 and CCND1 in CRC cell lines treated with Pyrvinium or vehicle control. Cells were treated, lysed in protein sample buffer and immunoblotted with the cognate antibodies for CTNNB1, CCND1 and GAPDH control.

As we have previously suggested that Pyrvinium exerts its biological effects through attenuating WNT signaling activity, we tested Pyrvinium's ability to inhibit WNT activity in CRC cell lines expressing a WNT-dependent reporter driving luciferase expression. Pyrvinium was able to reduce luciferase expression in a potent, dose-dependent manner (Fig 2A). Moreover, Pyrvinium also reduced the level of the WNT-dependent protein biomarker in these CRC cells, PYOGOPUS2 (Fig 2A). We next examined the expression of a number of endogenous WNT-regulated genes in CRC cell lines. In these cells, Pyrvinium also repressed the expression levels of the WNT-regulated genes *AXIN2*, *DIKKOPF-RELATED PROTEIN 1 (DKK1), CYCLIN-D1 (CCND1), CTNNB1* and *LEUCINE-RICH-REPEAT-CONTAINING G-PROTEIN RECEPTOR 5 (LGR5)* (Fig 2B), which collectively act as biomarkers of WNT–driven tumorigenesis in the intestinal epithelium [3], [12]–[14], [44], [48]–[50]. Consistent with the reduced transcription of these WNT biomarkers, Pyrvinium treatment decreased CTNNB1 protein levels in the nucleus of CRC cells (Fig 2C). Pyrvinium also reduced the steady-state protein level of CTNNB1 and CCND1 (Fig 2D). We note that the WNT target genes are not consistently inhibited by Pyrvinium in different CRC cell lines, and that these differences are likely due to the different mutational status of the individual cell lines- in which specific target genes might be regulated by other signaling pathways. Many WNT target genes have also been known to be regulated other multiple signaling pathways (for example, *CCND1* is also regulated by Notch [51]). Further, other WNT inhibitors have shown similar variation in distinct CRC cell lines [52]. We also note that the regulation of *CCND1* mRNA and protein are differentially regulated by Pyrvinium in two CRC cell lines (Fig2 B & D). This is likely a reflection between differences in steady-state levels of *CYCLIND1* mRNA and WNT protein, which is regulated by protein degradation in numerous ways, and as such here serves more as an indicator of decreased proliferation [53].

**Figure 3:**
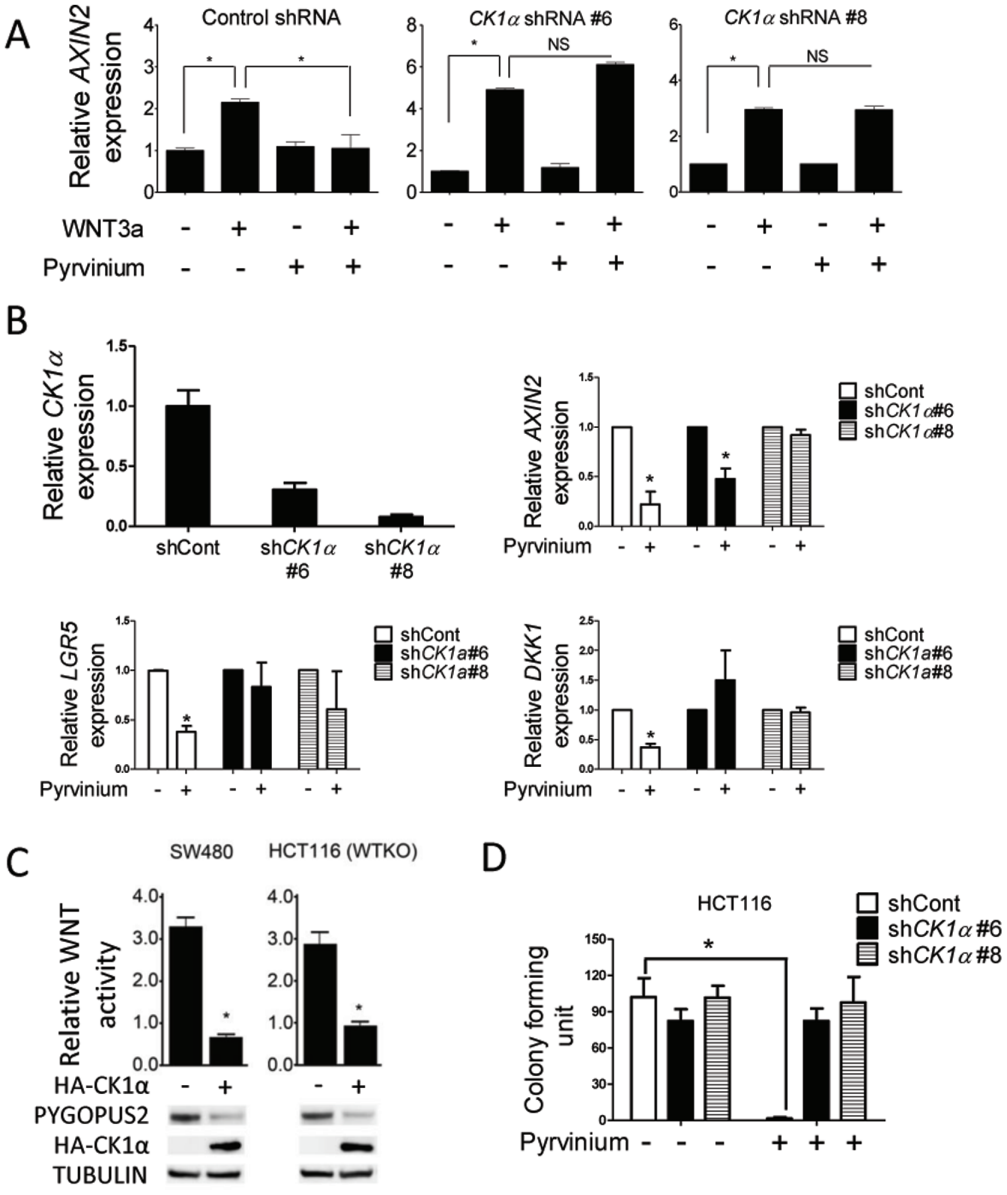
Pyrvinium inhibits WNT signaling in a CK1α dependent manner. **A**. The expression of the WNT target gene: *AXIN2* was determined from NIH 3T3 cells expressing control or *CK1α*-specific shRNAs. Cells were treated with control or WNT3a conditioned media with or without 100 nM Pyrvinium. *AXIN2* expression was quantified using real-time RT-PCR. Error bars indicate ± S.E.M of three experiments. *p<0.05. **B**. Pyrvinium suppressed WNT signaling biomarkers in a CK1α dependent manner. Upper left, *CK1α* expression was assessed by real-time RT-PCR in control shRNA or *CK1α* shRNA expressing HCT116 cells. The three other panels show control shRNA or *CK1α* shRNA infected HCT116 cells treated with or without 100 nM Pyrvinium for 24 hours and *AXIN2, DKK1 and LGR5* expression. Error bars indicate ± S.E.M of three experiments. *p<0.05. **C**. Overexpression of CK1α inhibits WNT reporter activity and decreases steady state levels of PYGOPUS2 in CRC cells. SW480 and HCT116 WTKO cells harboring the TOPflash reporter were transfected with a control plasmid or one expressing *HA-CK1α*. Immunoblots show decreased PYGOPUS2 levels for SW480 and HCT116 WTKO cells. Error bars indicate ± S.E.M of three experiments. *p<0.05. **D**. HCT116 stably expressing the indicated shRNA were treated with or without 100 nM Pyrvinium followed by determination of their colony forming capacity. Error bars indicate ± S.E.M of three experiments. *p<0.05.

To establish that Pyrvinium's inhibition of WNT activity is dependent on CK1α expression, we identified two independent *CK1α* specific shRNAs capable of reducing *CK1α* levels (data not shown and Fig S2). Pyrvinium potently repressed the expression of the WNT biomarker *AXIN2* in 3T3 cells stimulated with conditioned media containing WNT3a ligand, but did so in a manner that depended on *CK1α* expression (Fig 3A). We next established HCT116 cells stably expressing a control shRNA or one of these two *CK1α* specific shRNA (Fig 3B). Pyrvinium failed to suppress three WNT signaling biomarkers (Fig 3B) in cells with reduced *CK1α* levels, relative to cells expressing a control shRNA. Consistent with Pyrvinium's ability to enhance CK1α activity, ectopic expression of CK1α attenuated WNT reporter gene activity and reduced PYGOPUS2 levels in CRC cells (Fig 3C). Pyrvinium also failed to suppress the colony formation in CRC cells with reduced levels of *CK1α* relative to control CRC cells (Fig 3D), although such colonies were slightly smaller than those expressing control shRNA (see Fig S3). Taken together, these results suggest that Pyrvinium is able to significantly inhibit WNT-driven cell viability in CRC through CK1α.

### Pyrvinium has specificity for CK1 α in CRC cells

**Figure 4:**
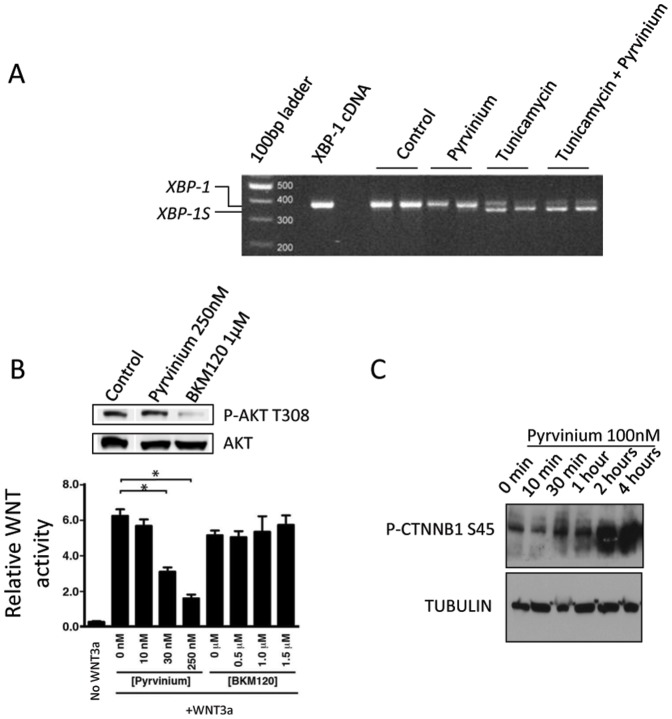
Inhibition of WNT signaling by Pyrvinium is independent of UPR or AKT inhibition. **A**. HCT116 cells under normal culture conditions were treated with vehicle control and either 100 nM Pyrvinium, 1 µg/mL Tunicamycin or a combination of 100 nM Pyrvinium and 1 µg/mL Tunicamycin for 24 h. cDNA from these cells was assessed for *XBP-1* mRNA splicing using *XBP-1* specific probes. XBP-1 full-length transcript produces a 380 bp PCR product, while the XBP-1S shortened splice variant produces a 350 bp PCR product. **B**. Pyrvinium inhibits WNT signaling at concentrations that does not block AKT activation. HEK 293 cells stably expressing a WNT responsive reporter gene were stimulated with or without WNT3a and the indicated concentrations of Pyrvinium or the pan-PI3K inhibitor BKM120. Error bars indicate ± S.E.M of four experiments. *p<0.05. As a control, we show that while BKM120 is capable of attenuating AKT activation using a phospho-specfic antibody to AKT [phospho-AKT (T308)], Pyrvinium does not inhibit AKT activation at concentrations sufficient to inhibit WNT signaling (Upper panels). Lysates from HEK 293 cells treated with indicated drugs were immunoblotted for phospho-AKT (T308) or total AKT, as indicated. **C**. SW480 cells were treated with 100 nM Pyrvinium for the indicated time points and S45 phosphorylated CTNNB1 levels, a known CK1α substrate, were detected by immunoblot.

We have previously shown that Pyrvinium binds directly, with high affinity, to CK1α to enhance its kinase activity, consistent with the results shown above. However, other groups have suggested alternate mechanisms underlying Pyrvinium's anti-cancer properties. One study suggested that Pyrvinium can inhibit CRC cell growth via inhibition of the unfolded protein response (UPR), which is often utilized by tumor cells for survival under low nutrient conditions [54]. Under normal nutrient conditions, 100 nM Pyrvinium was still able to inhibit CRC cell proliferation (Fig 1B) without influencing *X-BOX BINDING PROTEIN* (*XBP-1*) splicing (Fig 4A), a process commonly upregulated upon response to activation of UPR (such as that induced by Tunicamycin). Therefore, the Pyrvinium mediated repression of cell viability observed in CRC cells was not dependent on UPR repression. More recently, it has been suggested that Pyrvinium attenuates WNT activity by blocking activation of AKT, preventing GSK3β phosphorylation and its subsequent inactivation, which results in the phosphorylation and de-stabilization of β-CATENIN [55]. However, our data suggests that Pyrvinium at concentrations capable of attenuating WNT signaling do not block AKT activation (Figure 4B). Consistent with these latter findings, we recently determined Pyrvinium's activity using Ambit's scanMAX kinase profiling service. Utilizing 442 purified kinases (the largest commercially available kinase panel), the scanMAX kinase screen failed to detect inhibition of any of the kinases tested (data not shown). Significantly, this panel contained AKT1-3 isoforms and all known PI 3-kinase isoforms. Furthermore, consistent with Pyrvinium acting through CK1α, we show that the addition of Pyrvinium to CRC cells results in the time-dependent phosphorylation of a known CK1α substrate (Fig 4C). These latter results are consistent with Pyrvinium-mediated WNT inhibition acting via activation of CK1α to reduce CRC cell viability (Fig 1B and D).

### Pyrvinium inhibits intestinal adenomatous polyp formation in APC^min^ mice

**Figure 5:**
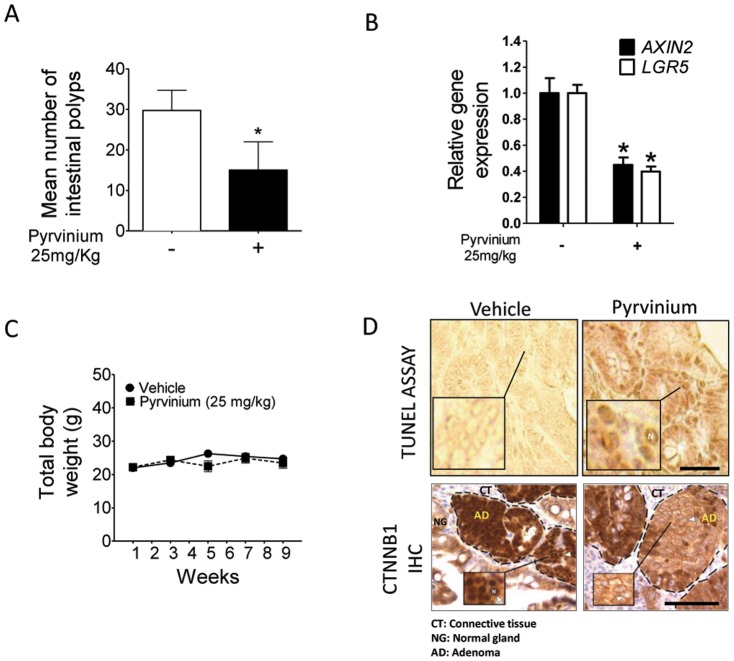
Pyrvinium reduces adenoma formation *in vivo*. **A**. Mean number of intestinal adenomas in Pyrvinium (25 mg/kg) or vehicle treated mice. Mice were treated via oral gavage once every 48 h for 10 weeks. Intestines were removed from euthanized mice, fixed in phosphate buffered formalin and then adenomatous polyps were quantitated. Error bars represent S.E.M, n = 12. *p<0.05. **B**. Expression levels of the WNT target genes *AXIN2* and *LGR5* in the intestinal epithelium of *APC^min^* mice chronically treated with vehicle control or 25 mg/Kg Pyrvinium. Total RNA from intestinal tissue sections of euthanized mice was converted to cDNA and subjected to real-time RT-PCR analysis. **C**. Mice treated with Pyrvinium for 10 weeks via oral gavage showed no significant weight loss in response to Pyrvinium treatment. **D**. Upper panels, TUNEL assays of intestinal sections from chronic Pyrvinium or vehicle treated *APC^min^* mice. Intestinal tissue sections were isolated from euthanized mice embedded in paraffin and subjected to an in situ TUNEL assay using the DeadEnd colorimetric TUNEL assay (N: nucleus). Lower panels, Immunohistochemistry showing CTNNB1 expression and localization in intestinal sections from Pyrvinium and vehicle treated mice. Mice were treated for 7 days. Intestinal tissues isolated from euthanized mice were fixed using buffered formalin, paraffin embedded and subjected to immunohistochemistry using an antibody for CTNNB1 (N: nucleus). Scale = 50 µm.

As Pyrvinium proved to be an efficient suppressor of WNT-regulated gene expression and CRC cell growth, we next determined Pyrvinium's ability to attenuate the pathological phenotype characteristic of FAP: adenomatous polyp formation. To accomplish this we treated *APC^min^* mice via oral gavage with 25 mg/kg Pyrvinium, or vehicle control, once every 48 h for 10 weeks. Pyrvinium treatment significantly inhibited polyp formation in *APC^min^* mice (Fig 5A). Consistent with this attenuation occurring through attenuation of WNT signaling, mice chronically treated with Pyrvinium exhibited a ∼50% reduction in *AXIN2* and *LGR5* expression in the colon epithelium (Fig 5B) relative to vehicle treated mice. This effect was achieved at a dose of Pyrvinium that did not produce any overt systemic toxicity, as evidenced by no reduction in body weight in treated mice over the course of the study (Fig 5C). Previous studies have shown that mice exhibit no toxicity at doses up to 60 mg/kg Pyrvinium (p.o) [56]–[59], above the effective dose used here. When assessed by *in situ* TUNEL assay Pyrvinium treated mice also displayed more regions of apoptotic DNA fragmentation relative to that of vehicle treated mice (Fig 5D). Pyrvinium treatment also inhibited the nuclear localization of CTNNB1 in the intestines of *APC^min^* mice (Fig 5D). Thus, Pyrvinium can efficiently disrupt intestinal hyperplasia *in vivo* through targeted inhibition of WNT signaling with high efficacy and limited toxicity.

## Discussion

The WNT-signaling pathway has a well-documented, important role in FAP and eventual CRC. Our study demonstrates that targeted WNT-pathway inactivation may be an effective strategy for treating *APC*-mutant driven intestinal neoplasms. Utilizing the FAP mouse model *APC^min^* mice, we tested whether or not targeted inhibition of WNT activity using Pyrvinium could decrease polyp numbers *in vivo*. We demonstrated that Pyrvinium is a potent inhibitor of cultured CRC cell growth and viability and has efficacy *in vivo* when orally administered to *APC^min^* mice. Pyrvinium is an FDA-approved drug and has been proven safe for use in humans at doses as high as 35 mg/kg without any toxic effects. Therefore, Pyrvinium is a prime candidate for rapid transition to clinical trials as a chemotherapeutic agent for treatment of polyposis in FAP patients. Intrinsic to this discovery is the broader implication that Pyrvinium and related WNT-inhibitors may comprise a new spectrum of potent FAP drugs.

As an anti-helminthic drug Pyrvinium has limited absorption into the bloodstream when orally administered [57], [60]. This limited bioavailability restricts the use of Pyrvinium as a treatment for CRC metastases and other extra-intestinal FAP tumors. This also may partially explain the inability of Pyrvinium to effectively inhibit tumor xenograft growth observed by other groups [54]. Conversely, since Pyrvinium only exerts its effect on the intended target tissue: the intestinal epithelium, there should be little if any off-target effects of such treatment, as seen with the documented pernicious cardiovascular effects associated with NSAIDs. Similar WNT-inhibitors that are more efficiently absorbed into the bloodstream could be developed and tested against extra-intestinal FAP-associated tumors. Although Pyrvinium itself has not been demonstrated to directly cause DNA damage [56], [57], it substantially enhanced the efficacy of the chemotherapy drugs 5-Flourouracil, Irinotecan and Oxaliplatin [54] (and data not shown). These factors make Pyrvinium a promising candidate for both FAP and CRC chemoprevention. Importantly, the use of Pyrvinium to treat FAP patients is expected to lack the cardiovascular and gastrointestinal side effects of NSAIDs.

*CK1α* expression is required for Pyrvinium to inhibit WNT-driven target gene expression and clonogenicity, consistent with Pyrvinium acting through CK1α to inhibit WNT signaling [36]. Interestingly, Pyrvinium appears to inhibit WNT signaling by increasing the phosphorylation of a number of factors, including ones downstream of *CTNNB1* like *PYGOPUS* [36]. Recent studies in *CK1α* conditional-knockout mice showed that loss of CK1α protein resulted in robust WNT-activation and enhanced intestinal epithelial cell proliferation [39]. Abrogated *CK1α* expression, after loss of *p53* expression, resulted in a more invasive CRC phenotype than with *p53* loss alone [39]. In addition, *CK1α* expression was suppressed in melanoma metastases and reduced the number of melanoma metastases when ectopically expressed *in vivo* [38]. In this and other studies *CK1α* expression and activation in CRC cells inhibited WNT-driven luciferase reporter and endogenous WNT-regulated gene expression (Fig 2A; Fig 3C). It has also been shown that p53-mediated repression of WNT-activity requires CK1α kinase activity [39].Together these results suggest that *CK1α* may have a pivotal anti-tumorigenic role in the intestinal epithelium *in vivo*. Whether targeted activation of CK1α is an effective strategy for CRC treatment *in vivo* has yet to be demonstrated and requires further study.

## Supporting Information

Figure S1 **Clonogenic assay of CRC cell lines.** Representative images from Fig 1D.
(TIF)

Figure S2 **Knockdown efficiency of** ***CK1α*** **shRNA in NIH 3T3 cells.** **A**. Cultured NIH 3T3 cells were infected with viruses containing either control or *CK1α* shRNAs. Cells were lysed 72 h after infection and CK1α and β-ACTIN protein detected using the cognate primary antibodies.
(TIF)

Figure S3 **Clonogenic activity of HCT116 cells is CK1α dependent.** Representative images from Fig 3D.
(TIF)
