# Supplementary figures and images for: Repurposing the FDA-Approved Pinworm Drug Pyrvinium as a Novel Chemotherapeutic Agent for Intestinal Polyposis
Source: PLoS One. 2014 Jul 8;9(7):e101969. doi: 10.1371/journal.pone.0101969 (PMC4086981; doi:10.1371/journal.pone.0101969)

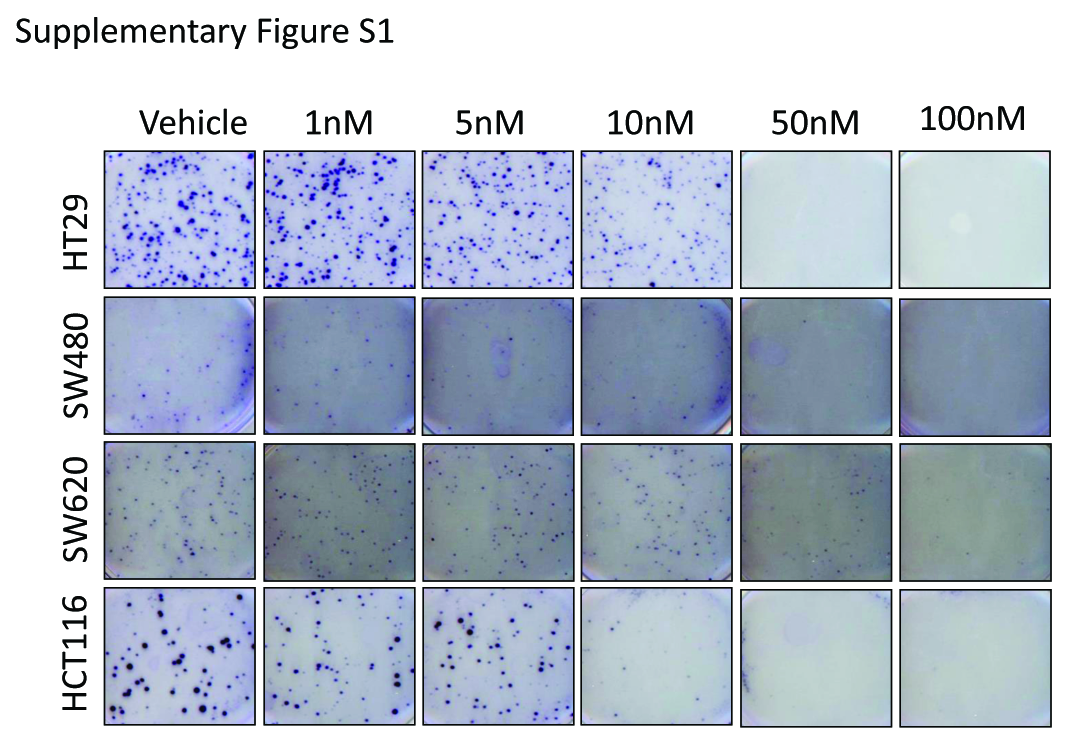

Supplement: Figure S1 — Clonogenic assay of CRC cell lines. Representative images from Fig 1D. (TIF) [file pone.0101969.s001.tif]

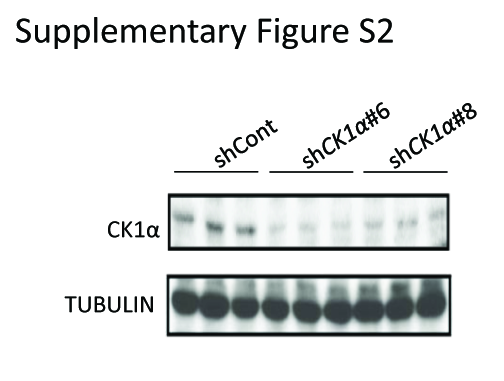

Supplement: Figure S2 — Knockdown efficiency of CK1α shRNA in NIH 3T3 cells. A. Cultured NIH 3T3 cells were infected with viruses containing either control or CK1α shRNAs. Cells were lysed 72 h after infection and CK1α and β-ACTIN protein detected using the cognate primary antibodies. (TIF) [file pone.0101969.s002.tif]

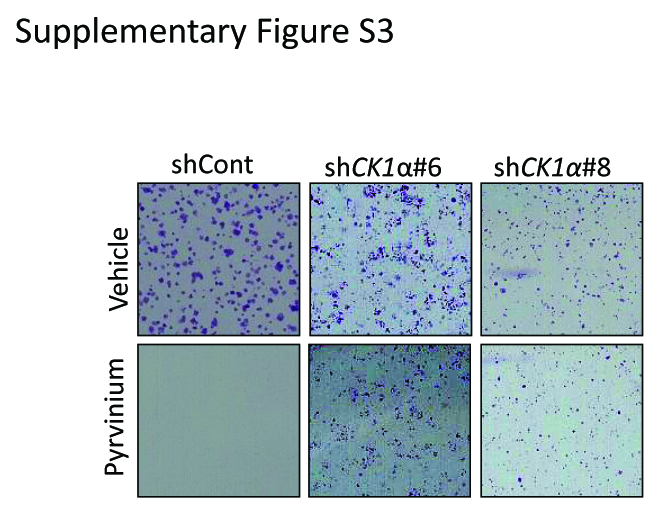

Supplement: Figure S3 — Clonogenic activity of HCT116 cells is CK1α dependent. Representative images from Fig 3D. (TIF) [file pone.0101969.s003.tif]
